# Palatal Abscess of Endodontic Origin with Extensive Radiolucency in Maxillary CBCT Imaging

**DOI:** 10.3390/diagnostics15172195

**Published:** 2025-08-29

**Authors:** Diana Marian, George Dumitru Constantin, Ademir Horia Stana, Ioana Elena Lile, Tareq Hajaj, Otilia Lavinia Gag (Stana)

**Affiliations:** 1 Department of Dentistry, Faculty of Dentistry, “Vasile Goldiș” Western University of Arad, 94-96 Revolutiei Blvd., 310025 Arad, Romania; marian.diana@uvvg.ro (D.M.); stana.otilia@uvvg.ro (O.L.G.); 2 Discipline of Clinical Practical Skills, Department I Nursing, Faculty of Medicine, Victor Babes University of Medicine and Pharmacy, 300041 Timisoara, Romania; george.constantin@umft.ro; 3 Department of Medicine, Faculty of Medicine, “Vasile Goldiș” Western University of Arad, 94-96 Revolutiei Blvd., 310025 Arad, Romania; stana.ademir@uvvg.ro; 4 Department of Propaedeutics and Dental Materials, Faculty of Dentistry, Victor Babes University of Medicine and Pharmacy, 2 Eftimie Murgu Sq., 300041 Timisoara, Romania; 5 Research Center in Dental Medicine Using Conventional and Alternative Technologies, Faculty of Dental Medicine, Victor Babes University of Medicine and Pharmacy of Timisoara, 9 Revolutiei 1989 Ave., 300070 Timisoara, Romania

**Keywords:** palatal abscess, endodontic infection, CBCT, cortical bone perforation

## Abstract

Palatal abscesses of endodontic origin are rarer than buccal ones due to maxillary anatomy. Their clinical appearance may resemble that of other palatal illnesses, complicating diagnosis and treatment. Prevention of problems requires early detection and endodontic treatment. A 26-year-old female patient presented with a 2 cm diameter palatal abscess, significant pulsatile discomfort, fever, and enlargement of the anterior hard palate. Clinical examination showed grade 1 mobility of the central and lateral incisors, percussion discomfort, and negative pulp vitality in the case of the lateral incisor. Cone-beam computed tomography (CBCT) showed two radiolucent lesions: a posterior cystic lesion near the first molar and an anterior lesion near the upper left lateral incisor. Palatal cortical bone puncture and soft tissue extension indicated the abscess origin. According to the clinical and imaging evaluation, the upper left lateral incisor had a persistent periapical lesion of endodontic origin that a palatal abscess with cortical bone perforation had exacerbated.

**Figure 1:**
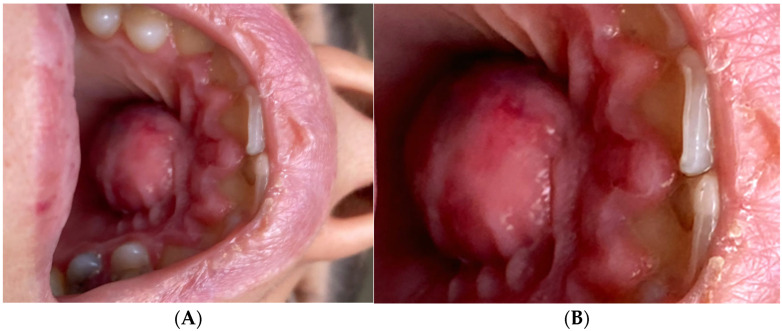
(**A**) Image of the anterior hard palate of a 26-year-old female patient. (**B**) A well-defined, dome-shaped, erythematous swelling is present on the anterior hard palate. The patient presented at our department with a palatal abscess approximately 2 cm in diameter, febrility, severe pulsatile pain which worsened during mastication, and excruciating palatal swelling with fluctuation on palpation. She reported difficulty eating and speaking, and noted that over-the-counter analgesics provided minimal relief. The patient’s chief complaint was severe palatal pain and swelling of 5 days’ duration, which began as mild palatal soreness and progressively worsened. She denied any history of trauma, recent dental treatment in the anterior maxilla, or similar past episodes. Her prior dental history was unremarkable, consisting of standard examinations and posterior tooth restorations only. She had no known systemic diseases, allergies, or medications that could influence healing or prognosis. The swelling had a fluctuant consistency on palpation, and there was no visible fistulous tract. Extraoral examination revealed no facial asymmetry or lymphadenopathy. Intraoral inspection confirmed a dome-shaped swelling on the anterior hard palate, measuring approximately 2 cm in diameter, with erythematous mucosa and fluctuant consistency. Endodontic assessment showed that teeth 21 and 22 elicited no response to the cold vi-tality test (Endo Ice, Coltene/Whaledent, USA), while adjacent teeth 11 and 23 responded within normal limits. On vertical percussion, teeth 21 and 22 were markedly tender, whereas the adjacent teeth were non-tender. Periodontal probing depths were within normal limits circumferentially, without isolated deep pockets. Grade I mobility was noted for teeth 21 and 22. No conventional panoramic, periapical, or occlusal radiographs were obtained prior to CBCT examination. Given the clinical presentation of significant palatal swelling and the need to accurately assess the three-dimensional extent of the lesion and its relationship to adjacent anatomical structures, CBCT was selected as the primary imaging modality. The scan revealed palatal cortical bone perforation, confirmed the primary endodontic origin of the lesion, and distinguished it from an additional radiolucent area in the posterior maxilla. These details, which could not be obtained with conventional radiography, were considered sufficient for both diagnosis and treatment planning in this case. The clinical and imaging assessment indicated a diagnosis of a persistent periapical lesion of endodontic origin related to the upper left lateral incisor, worsened by a palatal abscess with cortical bone perforation. Despite the presence of two separate radiolucent regions on the CBCT scan, only the lesion linked to the upper left lateral incisor exhibited palatal cortical perforation and soft tissue extension, signifying it as the principal origin of the palatal abscess. The other radiolucent region, although evident, was an independent, asymptomatic observation devoid of clinical indications of drainage or oedema. Clinically, teeth 21 and 22 demonstrated complete loss of pulp vitality on cold sensibility testing, marked tenderness to vertical percussion, and grade I mobility, while periodontal probing depths were within normal limits, excluding a primarily periodontal cause. The presence of a dome-shaped, fluctuant swelling on the anterior hard palate, together with localised tenderness on palpation, supported the presence of an acute inflammatory process. CBCT imaging confirmed a well-defined radiolucent lesion in the anterior maxilla, showing significant bucco-palatal expansion and thinning of the palatal cortical plate. Importantly, axial views revealed a distinct perforation of the palatal cortical bone directly adjacent to the apex of the upper left lateral incisor, consistent with the drainage pathway of a chronic periapical infection. The absence of similar cortical defects in the second radiolucent lesion in the posterior maxilla excluded it as the source of the palatal abscess. The combined evidence from sensibility testing, percussion response, swelling characteristics, and CBCT confirmation of cortical perforation and apical pathology established the definitive diagnosis of a persistent periapical lesion of endodontic origin at the level of the lateral incisor, complicated by a palatal abscess.

**Figure 2:**
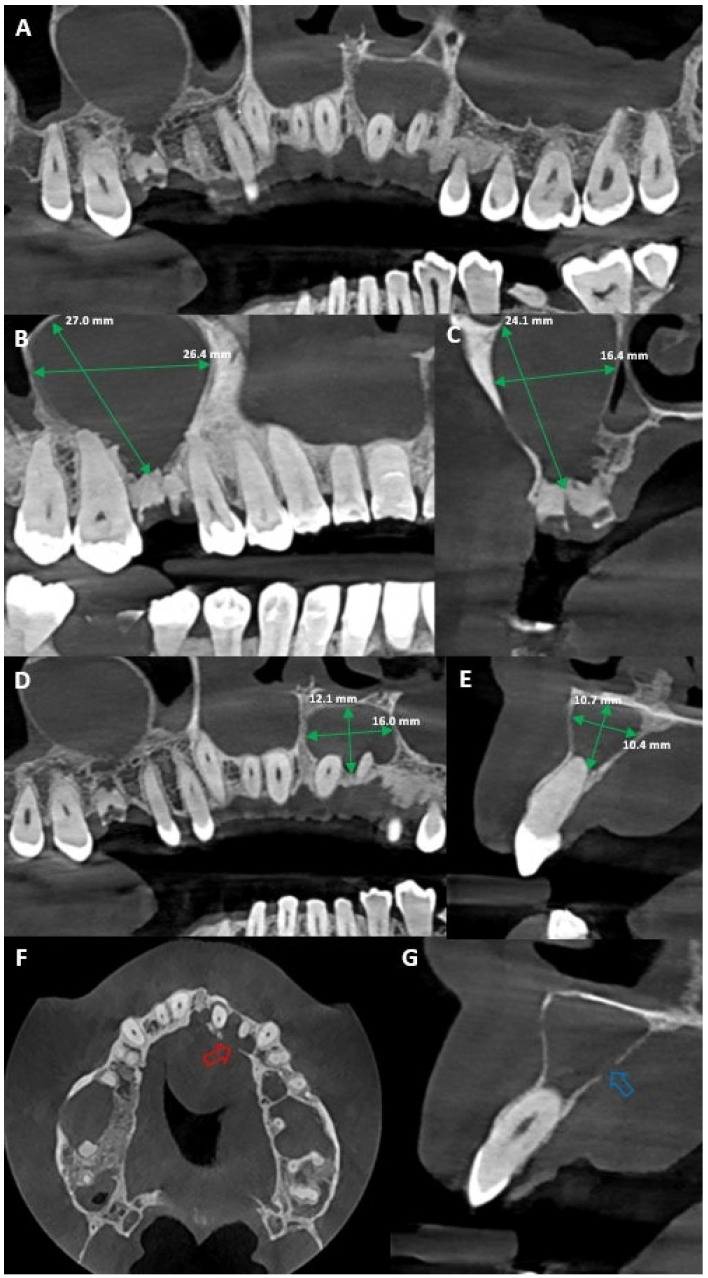
(**A**) Coronal CBCT reconstruction of the anterior and posterior maxilla. (**B**) Sagittal view reveals a clearly defined unilocular radiolucent lesion in the right maxillary region on the level of the first molar (27.0/26.4 mm). It shows both buccal and palatal expansion, as well as root displacement, consistent with a cystic lesion likely originating from the necrotic first permanent molar (tooth 16), which appears as a root remnant and presents a periapical radiolucency suggestive of chronic infection. (**C**) Sagittal view of the right first maxillary permanent molar lesion. (**D**) CBCT image reveals a second well-defined radiolucent lesion in the anterior maxilla (teeth 21 and 22) measuring approximately 12.1 mm by 16.0 mm, demonstrating significant bucco-palatal expansion and thinning of the palatal cortical plate, consistent with a chronic cystic process of odontogenic origin, caused by an endodontic lesion associated with the upper left lateral incisor. (**E**) Sagittal view of the lesion. (**F**) The axial CBCT view highlights a red arrow indicating a palatal cortical bone perforation (at level of tooth 22), which correlates clinically with the presence of a palatal abscess; this defect likely represents the drainage pathway of a chronic endodontic infection, most probably originating from the upper left lateral incisor, and confirms the extension of the lesion beyond the alveolar bone into the palatal soft tissues. (**G**) The axial CBCT section, with the blue arrow indicating the same palatal cortical bone perforation observed previously, confirming the pathway of infection through the palatal cortex; this axial perspective reinforces the presence of a drainage route associated with a chronic periapical lesion, most likely linked to the upper left lateral incisor, and supports the clinical finding of a palatal abscess. Similar cases of apical infection spreading to adjacent teeth have been reported, highlighting the importance of carefully assessing neighbouring areas when establishing a diagnosis [1]. It is well known that bone thickness may affect the drainage pathways of the odontogenic periapical abscess and the subsequent development of the sinus tract [2,3]. Emergency treatment was carried out to control the acute infection. Under local anaesthesia (2% lidocaine with 1:100,000 epinephrine), a linear incision was made over the most fluctuant area of the palatal swelling, followed by blunt dissection to allow drainage of purulent exudate. The abscess cavity was irrigated with sterile saline, and haemostasis was achieved. Systemic antibiotic therapy (amoxicillin/clavulanic acid 875 mg/125 mg every 12 h for 7 days) and analgesic/anti-inflammatory medication (ibuprofen 400–600 mg every 8 h) were prescribed to control infection and pain [4,5,6]. Root canal treatment in teeth 21 and 22 was initiated the following day, once the acute symptoms subsided and the patient was clinically stable, ensuring better tolerance of the procedure. Access opening was performed under rubber dam isolation, and working length was determined with an apex locator and confirmed radiographically. The canals were instrumented using a rotary NiTi system ProTaper Ultimate™ rotary file sequence (Dentsply Maillefer, Ballaigues, Switzerland) with copious irrigation using 5.25% sodium hypochlorite, followed by 17% EDTA for smear layer removal. Calcium hydroxide paste was placed as an intracanal medicament, and the teeth were temporarily restored.

**Figure 3:**
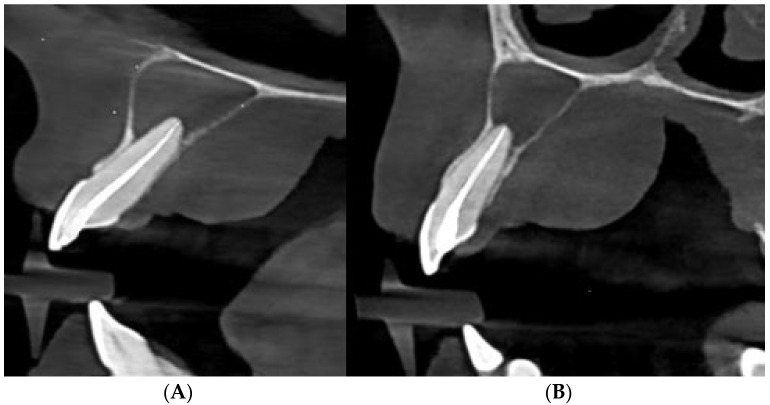
(**A**) CBCT sagittal view of the upper left central incisor (tooth 21) showing the postoperative obturation of the root canal and evidence of periapical bone rarefaction. (**B**) CBCT sagittal view of the upper left lateral incisor (tooth 22) showing root canal obturation and the persistence of a periapical radiolucency with palatal cortical perforation. At the follow-up appointment one week later, the patient was asymptomatic, and the swelling had completely resolved. The root canal was obturated using a bioceramic sealer and gutta-percha employing the hydraulic condensation technique. The access cavity was restored with composite resin to ensure a coronal seal. No postoperative periapical radiograph was obtained; instead, a CBCT scan was performed one week after obturation to provide a high-resolution baseline for follow-up, given the presence of two radiolucent lesions, marked bucco-palatal expansion, and a palatal cortical bone perforation. The patient was scheduled for a follow-up at 6 months to monitor healing of the periapical lesion and regeneration of the cortical plate. Regarding the right first maxillary permanent molar, it was considered non-restorable due to the significant coronal damage and the lack of a ferrule effect. The patient was referred to the oral surgery department for final management after it was determined that extraction with concurrent cystectomy was necessary. In line with reports highlighting persistent apical periodontitis caused by bacterial biofilms persisting in complex apical anatomies despite technically adequate treatment, our case underscores the importance of advanced imaging and tailored nonsurgical protocols to accurately localise the source of infection and guide effective management [7]. Apical periodontitis can be successfully treated and prevented with both nonsurgical and surgical endodontic therapies when they are performed in accordance with recognised clinical guidelines [8,9]. However, when apical periodontitis persists, further treatment, such as periradicular surgery, marsupialisation, decompression, and enucleation should be considered [8]. Similarly to reports on modified vacuum-assisted drainage for large periapical lesions, this case could demonstrate that conservative nonsurgical management can achieve complete resolution when effective decontamination and exudate removal are ensured [10]. Palatal abscesses of endodontic origin are relatively rare compared to buccal abscesses due to the anatomical structure of the maxilla, where the buccal side is more prone to infection spread [11]. Nevertheless, maxillary lateral incisors represent a notable exception. Owing to the palatal inclination of their root apices and the thinner palatal cortical plate in this region, they are more predisposed to palatal abscess formation. In addition, rare anatomical variations, such as an upper left lateral incisor with double roots and double root canals, can complicate diagnosis and treatment, requiring a customized approach [12]. They can be challenging to diagnose due to their similarity in presentation to other palatal lesions, such as periodontal abscesses, cysts, and neoplastic processes such as squamous-cell or mucoepidermoid carcinomas. Early diagnosis and treatment are crucial for preventing the systemic spread of the infection [13]. Therefore, correlating the occurrence of endodontic lesions progressing to abscesses with current clinical practices emphasises the necessity of updating and standardising treatment protocols to ensure optimal case management and reduce complication rates. Study of practitioners’ preferences can guide clinical decision-making, promoting the use of the most effective materials and techniques in everyday endodontic practice [14]. One important predictor of treatment failure is the existence of a preoperative cortical bone deficiency. In particular, individuals with palatal cortical bone deficiency are more likely to experience less healing [15]. Significant changes in lesion volume were noted throughout a 24-month follow-up period, highlighting the importance of long-term monitoring of patients undergoing root canal treatment for large cyst-like periapical lesions. This emphasises the necessity of continuous evaluation to guarantee efficient treatment of specific lesions [15,16]. Anamnesis and clinical examination are both used to make a diagnosis in endodontics. Together with complementary imaging tests, these data offers crucial details which aid in determining the prognosis and course of treatment [17,18]. In [19], a cone-beam computed tomography (CBCT) was performed to evaluate the palatal lesion three-dimensionally and identify the exact relationship with adjacent tooth roots, bone structures, and potential odontogenic causes. The role of CBCT in this case was pivotal: it allowed precise three-dimensional localisation of the lesion, detection of bucco-palatal bone destruction, and confirmation of the palatal cortical defect, while excluding other potential causes such as nasopalatine duct cysts, odontogenic keratocysts, or salivary gland tumours. Such details are challenging to obtain with conventional radiography alone. Compared to conventional 2D radiography, CBCT provided multiple distinct advantages in this case: accurate three-dimensional localisation of the lesion and confirmation of its odontogenic origin; comprehensive evaluation of bucco-palatal bone destruction with clear identification of the palatal cortical perforation; exclusion of root anomalies or fractures that could alter the treatment plan; and assessment of the lesion’s spatial relationship to critical anatomical structures such as the nasal floor and incisive canal. These imaging capabilities were crucial in confirming the diagnosis and justifying the choice of a conservative, nonsurgical approach. However, it must be emphasised that malignant tumours may radiographically mimic periapical lesions, in such atypical cases, caution and interdisciplinary collaboration are required [20]. In addition to CBCT, which played a pivotal role in the case reviewed here, emerging technologies such as artificial intelligence, augmented reality, and virtual reality offer further potential to enhance lesion detection, diagnostic accuracy, and treatment planning in complex endodontic infections [21]. In establishing the diagnosis, several potential differential diagnoses for an extensive palatal radiolucency were considered, including nasopalatine duct cyst, odontogenic keratocyst, benign fibro-osseous lesions, and salivary gland tumours such as mucoepidermoid carcinoma. The CBCT findings —specifically, the apparent association of the lesion with the apex of a non-vital tooth, the well-circumscribed radiolucent borders, and the detection of a palatal cortical bone perforation consistent with an inflammatory drainage pathway—strongly supported an endodontic origin. Management required a two-phase approach: immediate relief of acute infection by incision and drainage, followed by definitive source control through nonsurgical root canal treatment. The choice of a conservative endodontic approach was supported by the CBCT findings, which revealed no vertical root fracture and no proximity to critical anatomical structures that would necessitate surgical intervention. Long-term follow-up is essential in large cyst-like periapical lesions with cortical perforation. Healing potential is influenced by lesion size, host immune response, and the quality of the root canal obturation, with complete bone regeneration sometimes requiring up to 24 months. A key limitation of this case is the absence of six-month follow-up radiographs, as the patient has not yet attended the scheduled recall. Future imaging is essential for documenting healing and cortical bone regeneration. Additional limitations include the lack of conventional periapical radiographs and the need for extended follow-up to confirm complete bone regeneration. These factors restrict the ability to provide comprehensive radiographic comparisons over time. In summary, this case highlights the importance of incorporating CBCT into diagnostic protocols for atypical abscess presentations, as it enables precise lesion localisation, assessment of cortical bone integrity, and the development of customised treatment strategies. These findings reinforce the value of advanced imaging in improving prognosis and supporting evidence-based decision-making in both endodontics and oral medicine.

## Data Availability

The data are available upon request from the authors.
